# *Trichophyton mentagrophytes* ITS Genotype VIII/*Trichophyton indotineae* Infection and Antifungal Resistance in Bangladesh

**DOI:** 10.3390/jof10110768

**Published:** 2024-11-05

**Authors:** Mohammed Saiful Islam Bhuiyan, Shyam B. Verma, Gina-Marie Illigner, Silke Uhrlaß, Esther Klonowski, Anke Burmester, Towhida Noor, Pietro Nenoff

**Affiliations:** 1 Department of Dermatology and Venerology, Bangabandhu Sheikh Mujib Medical University (BSMMU), Dhaka 1000, Bangladesh; drsaifulib@bsmmu.edu.bd; 2 Nirvan & ‘In Skin Clinics’, Vadodara 390020, India; skindiaverma@gmail.com; 3 Labopart-Medizinische Laboratorien, D-04571 Rötha OT Mölbis, Germany; ginamarie.illigner1@gmail.com (G.-M.I.); s.uhrlass@labopart.de (S.U.); e.klonowski@labopart.de (E.K.); 4 Department of Dermatology, Jena University Hospital, Friedrich Schiller University, D-07747 Jena, Germany; anke.burmester@med.uni-jena.de; 5 Matador Skin Center, Dhaka 1000, Bangladesh; noortowhida@rocketmail.com

**Keywords:** dermatophytosis, Bangladesh, *Trichophyton indotineae*, *Trichophyton mentagrophytes* genotype VIII, terbinafine, voriconazole

## Abstract

*Trichophyton* (*T*.) *mentagrophytes* ITS genotype VIII, also known as *Trichophyton indotineae*, is a new species of the *T. mentagrophytes*/*T. interdigitale* complex and its first records, albeit under a different species name, are from the Indian subcontinent, Middle Eastern Asia, and West Asia*. T. mentagrophytes* genotype VIII (*T*. *indotineae*) has spread globally and has now been documented in over 30 countries. The aim of this study was to investigate the occurrence and proportion of terbinafine- and itraconazole-resistant isolates of *T. mentagrophytes ITS* genotype VIII (*T. indotineae*) in Bangladesh. This was part of an official collaborative project between IADVL (Indian Association of Dermatologists, Venereologists, and Leprologists) and Bangabandhu Sheikh Mujib Medical University (BSMMU), Bangladesh. Over a period of 6 months, ninety-nine patients of chronic recalcitrant tinea corporis were recruited from BSMMU hospital. Species identification was performed by fungal culture and morphological observation of the upper and lower surfaces of fungal colonies, as well as by using fluorescent microscopy. In addition, a PCR (polymerase chain reaction)-ELISA was performed to group the patients into those with the *T. mentagrophytes*/*T. interdigitale* complex. The internal transcribed spacer (ITS) gene was sequenced. Samples were tested for resistance to terbinafine and itraconazole by mutational analyses of the squalene epoxidase (*SQLE*) and the ergosterol 11B (*ERG11B*) genes. A total of 79/99 samples showed a positive culture. In 76 of these isolates, *T. mentagrophytes* ITS genotype VIII (*T. indotineae*) could be reliably identified both by culture and molecular testing. Resistance testing revealed terbinafine resistance in 49 and itraconazole resistance in 21 patients. Among these, 11 patients were resistant to both the antifungal agents. Mutations L393S, L393F, F397L, and F397I of the *SQLE* gene were associated with terbinafine resistance. Resistance to itraconazole could not be explained by mutations in the *ERG11B* gene. Infections with *T. mentagrophytes* ITS genotype VIII (*T. indotineae*) have become a public health issue with potentially global ramifications. About 62% of samples from Bangladesh showed resistance to terbinafine, making oral itraconazole the most effective drug currently available, although resistance to itraconazole and both terbinafine and itraconazole also exists.

## 1. Introduction

In recent years, dermatology outpatient departments (OPDs) in many parts of the world, especially in the Indian subcontinent, have been faced with a huge number of patients seeking recovery from dermatophytosis [1]. At the same time, treatment of dermatophytosis with the recommended dosing duration of conventional antifungal drugs has proven difficult due to chronic, persistent, and many novel clinical manifestations. Though the precise cause of this situation is not clear, host immunity, drugs, ecosystem (global warming), geographical region, cultural habits, and the pleomorphic character of the causative fungus have been considered as potential factors [2]. Among the seven genera of dermatophytes—*Arthroderma*, *Epidermophyton*, *Lophophyton*, *Microsporum*, *Nannizzia*, *Paraphyton*, and *Trichophyton*-*Trichophyton* (*T*.), *Microsporum*, *Epidermophyton*, and *Nannizzia* are the primary pathogens for humans [2]. When considering the history of epidemiological patterns of dermatophyte infection, it is striking that the spectrum of dermatophyte species has changed over time due to human migration and socioeconomic changes. From 1930 to 1950, *T. mentagrophytes* (today the *T. mentagrophytes*/*T. interdigitale* complex*)* was the main causative agent of tinea pedis and tinea corporis, while *T. rubrum* was very rare in Europe before the 1940s [3]. From 1950 onwards, *T*. *rubrum* spread over the next 30 years and remained the predominant species, followed by *T. mentagrophytes* as the causative agent for superficial fungal diseases worldwide, except scalp infections [4]. Some studies showed that *T. rubrum* was responsible for about 90% of cases of chronic dermatophytosis [5]. In 2008, *T. rubrum* was the leading causative dermatophyte species, accounting for 80% of cases [6]. However, in 2011, Sahai et al. in India reported a dramatic epidemiological shift of the dominant dermatophyte from *T. rubrum* to *T. mentagrophytes* [7]. Other Indian studies also described the predominant role of *T. mentagrophytes* [8,9].

*T. mentagrophytes*, the most polymorphic group among dermatophytes, is considered zoophilic and is responsible for highly inflammatory dermatophytosis when infecting human hosts. It is present worldwide and has spread independently of race and geography [4]. *T. interdigitale* was identified by sequencing of the internal transcribed spacer (ITS) region as a clonal anthropophilic derivative of *T. mentagrophytes* that causes non-inflammatory lesions. Recently, *T. mentagrophytes* was reported as the most common dermatophyte species in India and Iran [10,11,12]. These two sibling species represent a wide number of genotypes of the ITS region, but differentiation of these two species is difficult in practice and has been summarized into a *T. mentagrophytes*/*T. interdigitale* species group (TMTISG) with a high terbinafine resistance rate [13,14]. More than 10 ITS genotypes (TMTISG) have been identified with different geographical distribution and clinical pictures [14]. Among these, ITS genotype VIII was identified as a separate species causing chronic recalcitrant dermatophytosis in India [15,16]. This terbinafine and itraconazole resistance can be detected molecularly by sequencing the squalene epoxidase and the *Erg11* gene of the r-DNA [17,18]. In 2020, this terbinafine-resistant ITS genotype VIII was named as *Trichophyton* (*T.*) *indotineae* by Kano et al., as it was identified in one Indian and one Nepalese patient [19]. A previous case with the corresponding sequence was identified and reported from Australia in 2007 [20]. A sequence similar to that of *T. mentagrophytes* ITS genotype VIII (*T. indotineae*) was identified in a GenBank skin sample isolated from an Indian in 2004 (AB430471.1) [21]. The pathogen has spread to many countries of the Middle East, Europe, and North America. Although Bangladesh is a neighboring country of India with close foreign relations and its citizens travel irregularly between the two countries, no work on *T. mentagrophytes* ITS genotype VIII (*T. indotineae*) has been published from the country to date. Between 2008 to 2022, in fifteen published papers from different countries (none from Bangladesh), out of 100 reported cases of infection with *T. mentagrophytes* ITS genotype VIII (*T*. *indotineae*), 35% originated from India and 11% from Bangladesh [22,23,24,25,26,27]. The present study investigates the true extent of this new dermatophyte and its antifungal resistance pattern in Bangladesh.

## 2. Patients and Methods

Skin scraping samples were collected and investigated over a period of 6 months from ninety-nine patients with chronic recalcitrant tinea corporis from Bangladesh. These were patients with suspected dermatophytosis and with pronounced and highly inflammatory dermatophytia, which are characteristic of infections with *T. mentagrophytes* ITS genotype VIII (*T. indotineae*). No preliminary mycological diagnosis was performed prior to this study. Age, gender, and occupation of these patients were recorded. Duration and location of the dermatomycosis, as well as whether there was a relapse or recurrence of the symptoms, were also noted. Whether and which fixed-dose combination cream (FDC) was used and whether oral treatment with terbinafine, fluconazole, voriconazole, and itraconazole was used was also recorded. A table with the detailed data can be found in the Supplementary Materials.

**Figure 1:**
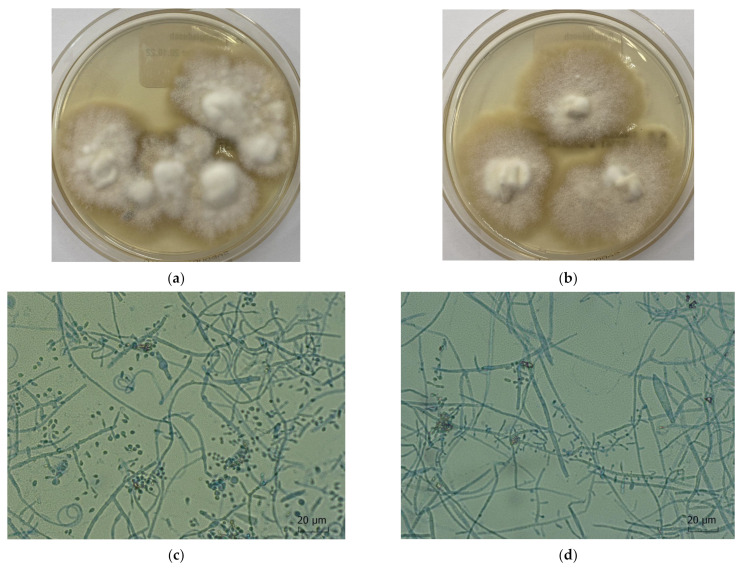
*T. mentagrophytes* genotype VIII (*T. indotineae*) on Sabouraud’s dextrose agar: (**a**) without cycloheximide; (**b**) with cycloheximide. (**c**,**d**) Microscopic image with micro- and macroconidia.

Species identification was performed by fungal culture and by morphological examination of the top and bottom surfaces of the fungal colonies, as well as by fluorescent microscopy (Figure 1). The Uniplex PCR-ELISA test was used to group the patients into those with the *T. mentagrophytes*/*T. interdigitale* complex [28]. Sequencing of the ITS gene was performed. Samples were tested for resistance to terbinafine and itraconazole by mutation analysis of the squalene epoxidase (*SQLE*) and *ERG11B* genes.

### 2.1. PCR for Determination of the Species from Skin Scraping Samples

DNA from the skin scrapings was extracted according to the manufacturer’s protocol using the QIAamp^®^ DNA Mini Kit (Qiagen, Hilden, Germany).

For species identification, PCR (polymerase chain reaction) was performed in which the dermatophyte DNA was amplified in the master cycler with specific primers. One primer of the primer pair was labeled with digoxigenin at the 5′ end to label the resulting PCR product with digoxigenin. The topoisomerase II gene was used for identification using the primer sequences described by Hsu et al. [29]. The master mix contained 2.5 mM MgCl_2_, 5* buffer with 400 mM Tris-HCl, 100 mM (NH_4_)_2_SO_4_, and 0.1% Tween-20 as well as 200 µM of each dNTP and the TaqDNA polymerase (Bio-Budget Technologies GmbH, Krefeld, Germany). The PCR mixture was prepared with a final volume of 30 µL. This is 6 µL master mix, 16.5 µL H_2_O, 0.75 µL Primer-U (20 µM primer unlabeled from biomers.net, Ulm, Germany), 0.75 µL Primer-D-Dig (primer labeled with digoxigenin, TIB Molbiol Syntheselabor GmbH, Berlin, Germany), and 6 µL DNA as template. An amount of 6 µL of water was used as a negative control and 6 µL of positive DNA as a positive control. The tubes were covered with mineral oil to prevent evaporation and contamination. The PCR program used included an initial denaturation at 95 °C for 5 min and 30 s, followed by 42 cycles: denaturation at 95 °C for 15 s, annealing at 63 °C for 20 s, extension at 72 °C for 90 s, and final extension at 72 °C for 7.7 min [30].

### 2.2. Visualization by PCR-ELISA for Direct Identification of Dermatophytes from Skin Scrapings

The samples were analyzed for dermatophyte DNA using a validated and standardized, in-house-developed enzyme-linked immunoassay (PCR-ELISA) [30]. Specific probes were used detecting the following relevant dermatophytes: *T*. *rubrum*, *T*. *violaceum*, *T. interdigitale*/*T. mentagrophytes*, *Microsporum* (*M*.) *canis*, *M*. *audouinii*, *T*. *benhamiae* (formerly referred to as *T.* anamorph of *Arthroderma benhamiae*), and *Epidermophyton floccosum*.

For the PCR-ELISA, the chemically denatured PCR product is hybridized after amplification with a biotinylated probe (also a sequence from the topoisomerase II gene) and bound to a streptavidin-coated solid phase. Unbound, nonspecific amplification products and DNA are removed by washing the microtiter plate. The positive reaction is indicated by color development after the addition of a peroxidase-conjugated anti-digoxigenin antibody and substrate (ABTS tablets, Roche Diagnostics Germany, Mannheim, Germany). The optical density (OD) is measured at a wavelength of 405 nm (TECAN Sunrise Photometer, Crailsheim, Germany) [28,30]. The PCR-ELISA is a culture-independent method that can be performed on all skin samples.

### 2.3. Identification of Dermatophyte Species by Sequencing of the Fungal DNA from Culture

ITS sequencing is used for species identification and is only performed on cultures after DNA isolation by the QIAamp^®^ DNA Mini Kit (Qiagen, Hilden, Germany). Sequencing was performed in all samples of fungal culture material. The identification of all isolated dermatophytes was confirmed by sequencing the ITS region of the ribosomal DNA (rDNA), mainly the regions ITS 1, 5.8S rRNA, and ITS 2 [31,32,33].

The required PCR amplification of a ~900 bp DNA fragment was performed using universal primers that bind to flanking panfungal sequence regions. The following gene sequences were used as probes for sequencing of the ITS region of the rDNA: V9G 5′-TTACGTCCCTGCCCTTTGTA-3′ and LSU266 5′-GCATTCCCAAACAACTCGACTC-3′ [34]. The PCR program used included an initial denaturation at 94 °C for 5 min, followed by 42 cycles: denaturation at 94 °C for 60 s, annealing at 63 °C for 60 s, extension at 72 °C for 60 s, and final extension at 72 °C for 10 min. Red HS Master Mix (Biozym Scientific GmbH, Hessisch Oldendorf, Germany) was used for the PCR. The DNA fragment was sequenced at the Microsynth Seqlab GmbH, Göttingen, Germany.

The sequence of each strain was compared with sequences of type strains from the databases. Based on the principle of similarity search (BLASTn search), individual strains were identified down to the species level using the validated Online Dermatophyte Database of the Westerdijk Fungal Biodiversity Institute (formerly Centraalbureau voor Schimmelcultures CBS), Utrecht, The Netherlands (https://wi.knaw.nl/ (accessed on 7 August 2024)). In addition, we compared sequences of our samples with those in the comprehensive database of the National Center for Biotechnology Information (NCBI) in Bethesda, MD, USA.

### 2.4. In Vitro Antifungal Susceptibility Testing of Trichophyton mentagrophytes Genotype VIII (Trichophyton indotineae) and Trichophyton rubrum

The antifungal susceptibility method used is an in vitro test of *T. mentagrophytes* and *T. rubrum* based on the work of Dr. Michel Monod, Lausanne, Switzerland [35]. For this purpose, an in-house test was developed. A 4-well culture plate from SPL Life Sciences Co. (Pocheon-si., Republic of Korea) is used, filled with Sabouraud’s dextrose agar (Sifin diagnostics GmbH, Berlin, Germany) with increasing terbinafine concentrations (0 µg/mL, 0.1 µg/mL, 0.2 µg/mL, 0.5 µg/mL in individual cases up to 16 µg/mL). One cm^2^ of culture surface is pre-textured in 1 mL of sterile water. For inoculation, 50 µL of the suspension is added to each well. Incubation is then carried out at 28° as previously described [25,36]. Fungal growth was examined after three to four days, and any growth was recorded as resistant. In vitro susceptibility to itraconazole was tested using the same breakpoint test with itraconazole concentrations of 0.125, 0.25, and 0.5 µg/mL. Sabouraud’s dextrose agar without antifungal agents was taken as the control. Based on epidemiological cut-off values (ECOFFs) from previous research, strains were classified as resistant or sensitive to terbinafine (epidemiological cut-off value or ECOFF of 0.125 µg/mL) and to itraconazole (ECOFF 0.25 µg/mL) [36,37]. Terbinafine and itraconazole were acquired from Sigma-Aldrich^®^, Merck KGaA, Darmstadt, Germany.

### 2.5. Squalene epoxidase Gene Analysis for Terbinafine Resistance Testing of Trichophyton mentagrophytes Genotype VIII (Trichophyton indotineae)

Fungal DNA was extracted from a fresh fungal culture of *T*. *mentagrophytes* ITS genotype VIII (*T*. *indotineae*) on Sabouraud’s dextrose agar (using a QIAamp^®^ DNA Mini Kit (Qiagen, Hilden, Germany). A square area of approximately 1.0 mm^2^ of the growing culture was used. The squalene epoxidase (*SQLE*) gene of the terbinafine-resistant clinical isolates was amplified by PCR with Red HS Master Mix (Biozym Scientific GmbH, Hessisch Oldendorf, Germany). The primer pair Tr*SQLE*-F1 (5′ ATGGTTGTAGAGGCTCCTCCC 3′) and Tr*SQLE*-R1 (5′ CTAGCTTTGAAGTTCGGCAAA 3′) was used, and chromosomal DNA served as the template [34] (initial denaturation: 5 min 95 °C/40 cycles/30 s 95 °C/30 s 60 °C/60 s 72 °C/final elongation 5 min 72 °C). The resulting PCR product with a length of approximately 1300 bp was sequenced at the Microsynth Seqlab GmbH, Göttingen, Germany. The sequences were aligned and screened for missense mutations using MEGA version 10.0.5 [38,39].

### 2.6. Mutation Analysis by PCR Using the DermaGenius^®^ Resistance Multiplex RT-PCR

The DermaGenius^®^ Resistance multiplex RT-PCR (PathoNostics, Maastricht, The Netherlands) is a terbinafine resistance test directly from the native sample. The test detects mutations in the squalene epoxidase gene as well as relevant *Trichophyton* strains [40,41,42,43]. The PCR kit used consists of ready-to-use, optimized mixtures of target-specific primers and probes for the detection and identification of the most common and clinically relevant dermatophyte species. It is based on real-time PCR technology, enabled by fluorescent probes present in the mixtures. Detection is enabled during amplification and melting curve analysis on a real-time PCR instrument that can detect fluorescence in green, yellow, orange, and red detection channels. If the melting temperature is below 64 °C, the sample has a mutation at 393 or 397 and terbinafine resistance is present. If the melting temperature is above 65 °C, the isolate does not have a mutation at 393 or 397 and there is no terbinafine resistance. DNA extracts from nail, hair, and skin material served as input material [44]. All 99 samples were tested, and the following mutations in the squalene epoxidase gene were detected: Leu393Phe, Phe397Leu, Leu393Ser, Phe397Ile, and Phe397Val.

### 2.7. Sequencing of the Erg11B Gene

First, the primers from the publication by Burmester et al. [18] were tested to investigate whether they could be used to sequence the suspected region of mutations that could be associated with azole resistance. The reverse primer was recalculated for a product length of 550 bp. This resulted in, among other things, the self-named primer *Erg11B*R1.

Primer *Erg11B*R1 (rev): 5′ AATAGTCAGTTGGCGGCACA 3′.

Primer Tm*Erg11B*F3 (fwd): 5′ GCCCACATGATGATTGCTCTTC 3′ [18].

The method used itself proved to be successful at a DNA concentration of up to 20 ng/µL. The self-created primers and PCR program delivered the expected sequences. The PCR program used included an initial denaturation at 95 °C for 5 min, followed by 35 cycles: denaturation at 95 °C for 60 s, annealing at 60 °C for 60 s, extension at 72 °C for 60 s, and final extension at 72 °C for 10 min. Red HS Master Mix (Biozym Scientific GmbH, Hessisch Oldendorf, Germany) was used for the PCR. DNA fragments were sequenced at the Microsynth Seqlab GmbH, Göttingen, Germany.

### 2.8. Phylogenetic Tree According to ITS-rDNA and tef1-α

The phylogenetic tree according to ITS-rDNA and *tef1-α* was constructed using the maximum likelihood method and the bootstrap method, rooted by *T. quinckeanum*. Evolutionary analyses were performed in MEGA X (version 10.1.6) [39,45]. Table 1 lists the genotypes and strains used for the dendrogram creation with the respective NCBI accession numbers.

### 2.9. Deposition of the Sequences in Gene Databases

The ITS gene sequences of a selection of five of the 79 strains/isolates of *T*. *mentagrophytes* ITS genotype VIII (*T. indotineae*) and one *T. rubrum* strain are deposited at the database of the National Center for Biotechnology Information (NCBI) in Bethesda, MD, USA (Table 2). In addition, the sequences of the selected strains were also deposited for translation elongations factor 1-α (*tef1-*α). The primers EF-DermF 5′ CACATTAACTTGGTCGTTATCG 3′ and EF-DermR 5′ CATCCTTGGAGATACCAGC 3′ were used with the PCR program (initial denaturation at 95 °C for 5 min, followed by 35 cycles: denaturation at 95 °C for 30 s, annealing at 58 °C for 30 s, extension at 72 °C for 60 s, and final extension at 72 °C for 5 min) [31].

### 2.10. Ethics Statement and Patient Informed Consent

The authors confirm that the ethical policies of the journal have been adhered to. No ethical approval was required as the research in this article was related to micro-organisms. All persons gave their informed consent prior to their inclusion in the study.

## 3. Results

### 3.1. Patients Data

**Figure 2:**
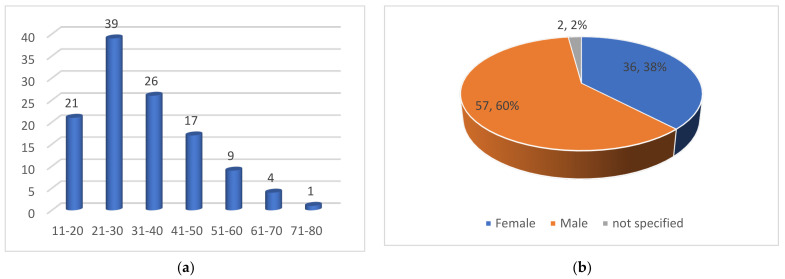
(**a**) Age distribution of patients with *T. mentagrophytes*/*T. interdigitale* complex (n = 95); (**b**) gender distribution of patients with *T. mentagrophytes*/*T. interdigitale* complex (n = 95).

Of the 99 samples, 95 strains of the *T. mentagrophytes*/*T. interdigitale* complex and 4 *T. rubrum* were identified by PCR and/or cultivation. To consider the clinical data, we focused on the evaluation of the 95 patients with the *T. mentagrophytes*/*T. interdigitale* complex. Of these patients, 54 were under 30 years of age and 41 were over 30. The affected individuals were 36 women, 57 men, and in two cases the gender was not specified (Figure 2a,b).

Of these 95 patients, 33 worked as housewives, 25 were students, 19 were in services, five worked as businessmen, three as peasants, two as industry workers, two as teachers, and one each as a retired person, unemployed person, shopkeeper, tailor, laborer, and in government service (Table 3).

**Figure 3:**
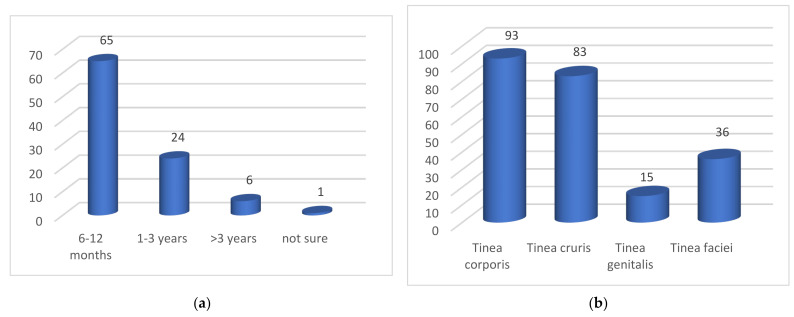
(**a**) Duration of illness before participation in the study (n = 95); (**b**) localization of tinea (multiple answers possible).

Before these patients took part in the study, they had suffered from skin diseases in 65 cases for 6–12 months, in 24 cases for 1–3 years, and in six cases for over 3 years. In one case, the affected person was not sure (Figure 3a). The location of the tinea can be divided into tinea corporis (includes trunk, buttocks, legs, arms), tinea cruris, tinea genitalis, and tinea faciei. A total of 93 patients reported tinea corporis, 83 tinea cruris, 15 tinea genitalis, and 36 tinea faciei. Multiple answers were possible (Figure 3b).

Previous treatment with fixed-dose combination creams (FDCs) containing clobetasol propionate or other corticosteroids with antifungal and antibacterial agents was noted and broken down. Approximately 33 patients used clobetasol + ofloxacin + ornidazole + terbinafine, 20 econazole + triamcinolone, 14 miconazole + hydrocortisone, six injections with triamcinolone, six clobetasol, two betamethasone, one mometasone, and one triamcinolone. In nine cases it was not clear whether treatment with FDC creams had taken place, in six cases “no” was stated, and in one case “yes”. Multiple preparations could be stated (Table 4).

**Figure 4:**
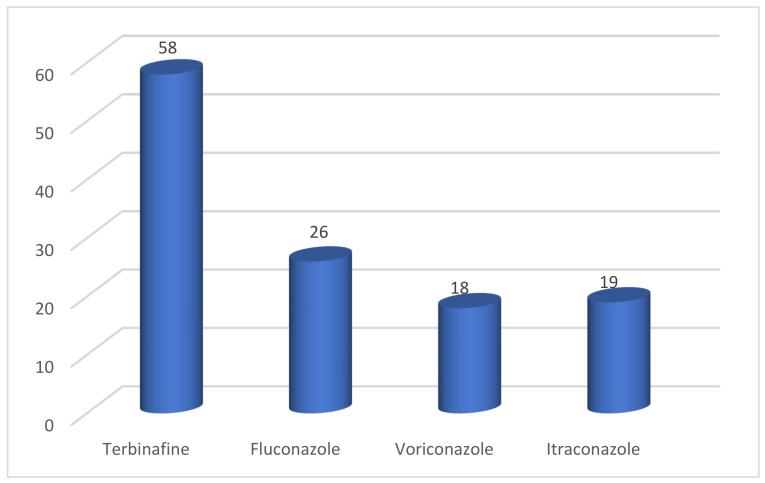
History of previous oral treatment. Specifying multiple preparations was possible.

Prior oral treatment with terbinafine, fluconazole, voriconazole, and itraconazole was noted, and it was possible that more than one antifungal agent was administered. Approximately 58 patients were treated with terbinafine and 12 were unsure if they had received this medication. Twenty-six patients were treated with fluconazole and 13 were unsure if they had received it. Eighteen patients were treated with voriconazole and 13 patients were unsure if they had received this medication. Nineteen patients were treated with itraconazole and 11 patients were unsure if they had received it (Figure 4).

### 3.2. Dermatophyte Detection by Culture and/or PCR

Dermatophytes were detected in 79 (78%) of 99 samples by both culture and PCR. The following dermatophytes were found: *T. mentagrophytes*/*T*. *interdigitale* (TM/Tinter), 76/79 (96.2%), and *T. rubrum*, 3/79 strains (3.8%).

### 3.3. Identification of Fungal Species and Genotypes by Sequencing of the ITS and the tef1-α Region of the rDNA

Since it was not possible to distinguish between *T*. *interdigitale* and the *T*. *mentagrophytes* complex using PCR-ELISA, the ITS region of the rDNA gene was sequenced. The sequencing focused exclusively on the cultural growth of the fungal isolates. Based on this sequencing, we were able to show that all *T. mentagrophytes* strains found belong to the *T. mentagrophytes* ITS genotype VIII (*T*. *indotineae*) [46].

**Figure 5:**
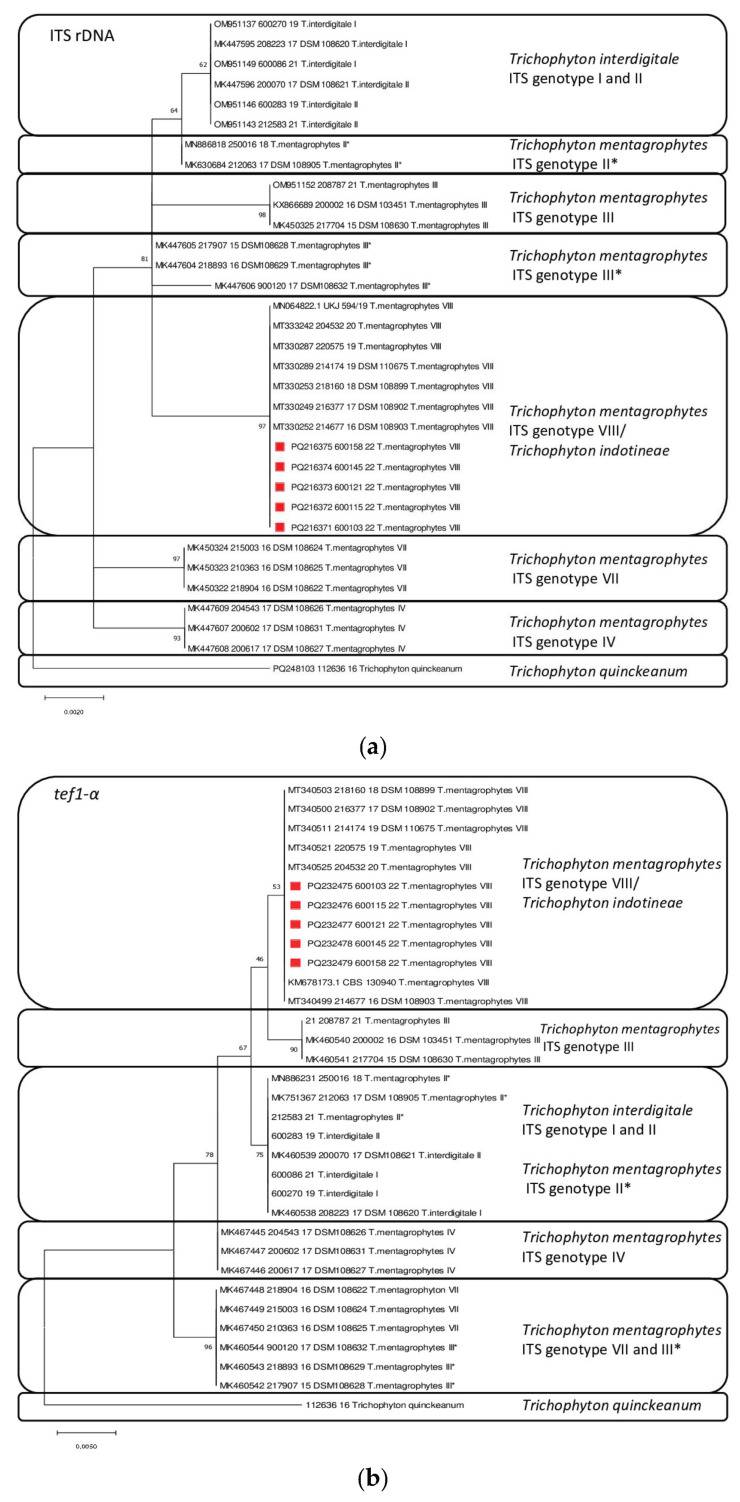
(**a**) Phylogenetic tree according to ITS-rDNA. Statistical method: maximum likelihood; test of phylogeny: bootstrap method; no. of bootstrap replications: 1000; red square: sequences from the Bangladesh study, rooted by *Trichophyton quinckeanum*. Evolutionary analyses were conducted in MEGA X (Version 10.1.6). III* is a subgroup of genotype III. (**b**) Phylogenetic tree according to tef1-α. Statistical method: maximum likelihood; test of phylogeny: bootstrap method; no. of bootstrap replications: 1000; red square: sequences from the Bangladesh study, rooted by *Trichophyton quinckeanum*. Evolutionary analyses were conducted in MEGA X (Version 10.1.6). III* is a subgroup of genotype III.

Phylogenetic trees of the *T. mentagrophyte ITS genotype* VIII (*T. indotineae*) strains from Bangladesh were constructed. For comparison, the most important genotypes of the *T. mentagrophytes*/*T. interdigitale* complex were included (Figure 5a,b).

A clear differentiation of *T. mentagrophytes* ITS genotype VIII (*T. indotineae*) from the other genotypes is possible both with regard to the ITS regions of the rDNA and the *tef1-α* gene.

### 3.4. Antifungal Resistance Testing and Point Mutation Analysis of Trichophyton mentagrophytes ITS Genotype VIII (Trichophyton indotineae)

#### 3.4.1. Antifungal Resistance Testing

**Figure 6:**
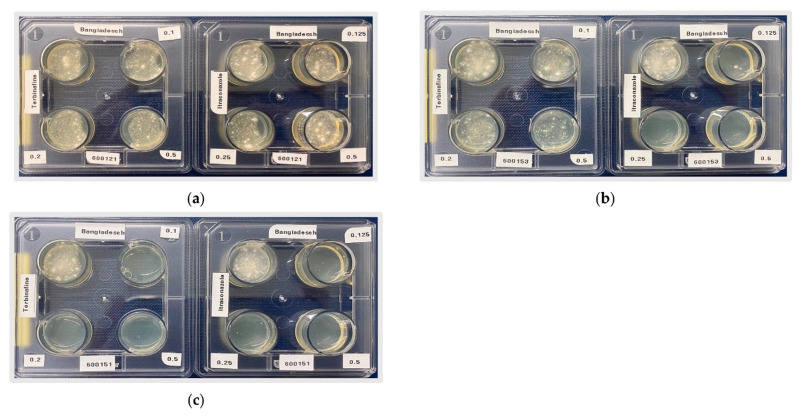
In-house breakpoint resistance testing of *T. mentagrophytes* ITS genotype VIII (*T. indotineae*) against terbinafine (**left**) and itraconazole (**right**). No visible growth in the well corresponds to sensitivity. Visible growth in the well means resistance: (**a**) strain is resistant to both terbinafine (MIC > 0.5 µg/mL) and itraconazole (MIC > 0.25 µg/mL); (**b**) strain is resistant to terbinafine (MIC > 0.5 µg/mL) and sensitive to itraconazole (MIC < 0.1 µg/mL); (**c**) strain is sensitive to terbinafine (MIC < 0.1 µg/mL) and itraconazole (MIC < 0.1 µg/mL).

Resistance testing of the 76 strains of *T. mentagrophytes* ITS genotype VIII (*T. indotineae*) using the breakpoint agar dilution method on Sabouraud’s dextrose agar containing terbinafine or itraconazole at different concentrations revealed terbinafine resistance in 49 and itraconazole resistance in 21 samples. Of these, 11 of 76 samples were resistant to both antifungal agents (Figure 6, Table 5).

#### 3.4.2. Mutation Analysis of Squalene Epoxidase

The sequences were evaluated using multiple sequence alignment. This reveals the point mutations that led to various protein replacements at positions 393, 397, 429, 436, and 448.

Sequencing of the *SQLE* gene showed various point mutations. The mutation Phe397Leu (F397L) is the most common with 20 strains (26%). There are three different point mutations that cause this amino acid exchange. There was a base exchange from TTC to TTA in four samples, an exchange from TTC to CTC in 14 samples, and one exchange from TTC to TTG. This is followed by Leu393Ser (L393S) from TTA to TCA with 20 strains (26%) and Ala448Thr (A448T) from GCT to ACT with 17 (22%). Ser436Ala (S436A) from TCC to GCC was found in five cases (7%) and Phe397Ile (F397I) from TTC to ATC, Leu393Phe (L393F) from TTA to TTC, and Asn429Asp (N429D) from AAC to GAC with 1.4% each. Double mutations in F397L (TTC to TTA) and A448T (GCT to ACT) occurred in four cases.

To illustrate the connections between mutations and resistance, these results were compared (Table 6).

Based on these results, 11 strains with mutations resistant to both terbinafine and itraconazole were detected among the samples. Two isolates had the mutation L393S, three isolates had F397L, two more isolates had the mutation S436A, and one isolate had the mutation A448T. Three isolates with the double mutation F397L and A448T were resistant to both terbinafine and itraconazole. About 38 strains with mutations were resistant to terbinafine and sensitive to itraconazole. Seventeen had the mutation L393S, 17 had the mutation L393S, and one each had the mutations S436A, F397I, and L393F. One isolate with the double mutation F397L and A448T was resistant to terbinafine and sensitive to itraconazole. Ten isolates with the A448T mutation were the only ones that were sensitive to terbinafine and resistant to itraconazole. Six isolates with the A448T mutation were sensitive to terbinafine and sensitive to itraconazole. In total, 10 strains with mutations were sensitive to terbinafine and sensitive to itraconazole. This includes eight A448T mutations, one L393S, two S436A, and one N429D. The remaining seven samples had no mutations (wild strains).

#### 3.4.3. Mutation Analysis by RT-PCR

Ninty-nine samples were examined using the DermaGenius^®^ Resistance multiplex RT-PCR. A total of 61 mutations were found, all of which can be assigned to the *T. interdigitale*/*T. mentagrophytes* complex. No mutations were found in the remaining 38 samples. Of these, 34 pathogens could be assigned to the *T. interdigitale*/*T. mentagrophytes* complex, and the remaining four pathogens to *T*. *rubrum* (Table 7).

Fourteen additional samples were identified using PCR that could not be sequenced previously. However, the DermaGenius^®^ PCR Resistance Kit detects fewer mutations than the mutation analysis of squalene epoxidase and therefore does not find the mutations Ser436Ala, Ala448Thr, and Asn429Asp. These are therefore always identified as sensitive.

#### 3.4.4. Sequencing of the Erg11B Gene

The mutations in the *Erg11B* gene were also analyzed by sequencing using multiple sequence alignment. Point mutations were identified which led to different protein exchanges at positions 441, 443, 444, and 445.

There were two different exchanges at position 441. In four samples the substitution Asp441Tyr (D441Y) was detected and in one sample the substitution Asp441Gly (D441G). The mutation Gly443Glu (G443E) was detected at position 443 in two isolates and the mutation Gly443Arg (G443R) in another. The largest number of mutations was found at position 444. Most of the mutations—in 30 samples—were identified as Tyr444His (Y444H). Furthermore, the base exchange Tyr444Cys (Y444C) was detected in five samples and Tyr444Ser (Y444S) in four samples. This position has the largest proportion of mutations examined at around 76%. Two-point mutations were found at position 445. Three isolates had the mutation Gly445Ser (G445S), and one isolate had the substitution Gly445Asp (G445D). A total of 51 point mutations were identified, corresponding to 65% of all samples.

The associations between mutations in the *Erg11B* gene and resistance to itraconazole are summarized in Table 8.

One isolate that was resistant to itraconazole in the breakpoint test had the Gly443Glu mutation. Two itraconazole-resistant samples had the Tyr444His mutation, and another two samples had the Tyr444Cys base change. The remaining itraconazole-resistant samples had the Gly445Ser mutation. Overall, 14% of samples with a mutation were resistant to itraconazole. However, fifteen samples without mutations in the *Erg11B* gene were also resistant to itraconazole in vitro. Thirteen isolates without the *Erg11B* gene mutation were not resistant to itraconazole. Seven isolates showed both itraconazole resistance and a mutation. Thirteen samples had neither itraconazole resistance nor a mutation. Fifteen samples showed resistance to itraconazole but no mutations. This meant that 51 samples could be classified as mutants, 28 samples as non-mutants, 22 samples as resistant, and 57 samples as sensitive (Table 9).

The Ala448Thr mutation in the *SQLE* gene is also associated with itraconazole resistance. A comparison between mutations in the *Erg11B* gene and Ala448Thr mutation in the *SQLE* gene is shown in Table 10.

Of 51 isolates with a mutation in the *Erg11B* gene, only one also had the Ala448Thr mutation in the *SQLE* gene. Of the 19 isolates with this mutation, 18 samples did not have a mutation in the *Erg11B* gene. However, of the isolates without a mutation in the *Erg11B* gene, 18 had the Ala448Thr mutation in the *SQLE* gene. The remaining ten isolates had neither a mutation in the *Erg11B* gene nor the Ala448Thr mutation in the *SQLE* gene.

## 4. Discussion

### 4.1. Fungal Infections in Bangladesh

There are few studies and case reports on invasive and superficial fungal infections in Bangladesh. Superficial mycoses are very common, with *T*. *rubrum* being the predominant etiological agent (80.6%). To date, no epidemiological studies on the occurrence of dermatophyte infections have been conducted in Bangladesh. Until a few years ago, *T. rubrum* was the main pathogen of superficial fungal infections in Bangladesh, but also in India and the world [47]. In India, there are now various epidemiological studies showing a change in the main pathogens of dermatophytoses from *T. rubrum* to the *T. mentagrophytes*/*T. interdigitale* complex [48,49]. The patients in this study were selected based on their clinical picture. That is, patients with pronounced dermatomycoses on the trunk, groin, and face were selected and included in the mycological examination.

### 4.2. Clinical and Anamnestic Patient Data

More than half of the patients with superficial fungal infections caused by *T. mentagrophytes* ITS genotype VIII were men. Most patients were young, between 10 and 40 years old. There were also older patients. The medical history did not provide any significant evidence of a connection with occupational activity. A high proportion of patients work in the home. In the majority of patients, tinea had been present for more than 6 months. The most common manifestation of mycosis caused by *T. mentagrophytes* ITS genotype VIII was tinea corporis, followed by tinea cruris and tinea genitalis. The frequent occurrence of tinea faciei was also typical. It is not surprising that tinea corporis accounts for the largest proportion, as this mycosis involves the torso, buttocks, legs, and arms. Tinea cruris is the typical site of infection in *T. mentagrophytes* ITS *genotype* VIII (*T. indotineae*) and, although it only affects the groin area, it has been described in 83 of 99 cases. The suspected connection between the occurrence of therapy-refractory tinea caused by *T. mentagrophytes* ITS genotype VIII and the misuse of strong topical corticosteroids in so-called combination or cocktail creams (FDCs) is supported by the history of the preparations used so far. A large proportion of patients used clobetasol in combination with antibiotics and antimycotics, but various other steroid creams were also used as an alternative. More than half of the patients had previously received oral antimycotic therapy with terbinafine. In addition, fluconazole, voriconazole, and itraconazole were used.

Ten terbinafine-sensitive cases (patient nos. 13, 28, 30, 36–38, 73, 77, 78, 93, 97) had a history of taking oral terbinafine without itraconazole or voriconazole and experienced relapse. It is not absolutely necessary that patients who have previously received terbinafine/itraconazole therapy also develop terbinafine resistance in vitro. This is especially true since *T. mentagrophytes* ITS genotype VIII (*T. indotineae*) strains can be acquired with or without terbinafine resistance through transmission from other people. Repeated relapses and treatment failure with oral antimycotics are typical for *T. indotineae* infections. These repeated relapses are certainly not only due to antimycotic resistance but are also due to the virulence and altered biological behavior of this new dermatophyte species.

### 4.3. Pathogen Identification

The pathogen identification or genotyping was based on the sequencing of the ITS region of the dermatophyte DNA. The sequences were identified via the NCBI BLAST. All 76 samples identified as *T. mentagrophytes* could be clearly assigned to genotype VIII. Since only two different *Trichophyton* species were found in the present study, the morphological evaluation was unproblematic. Despite morphological differences within a species, all fungal strains could be correctly identified by culture. Three of 79 isolates had the reddish-brown back of the dermatophyte colony, which enabled identification as *T. rubrum* and was confirmed by sequencing.

Using PCR-ELISA, all 99 skin scraping samples were reactive to a dermatophyte. The *T*. *mentagrophytes*/*T*. *interdigitale* complex was found in 95 of 99 samples (95.96%) using PCR-ELISA. DNA from *T*. *rubrum* was found in four samples (4.04%). However, the sequencing was not carried out from the fungal DNA extracted directly from skin scrapings, but rather, as shown above, from fungal culture material of the 76 cultured strains of the *T*. *mentagrophytes*/*T*. *interdigitale* complex, all of which were confirmed as *T. mentagrophytes* ITS genotype VIII (*T*. *indotineae*). Ultimately, it must be assumed that all samples that are positive for *T*. *mentagrophytes*/*T*. *interdigitale* by PCR-ELISA must be counted as belonging to the new species *T*. *indotineae*.

The mycological diagnosis of dermatophyte infection in the study was based on the routine procedure for dermatophyte detection in the Moelbis laboratory. A step-by-step diagnosis is carried out, starting with fluorescence microscopic preparation and cultural fungal detection. The diagnosis is supplemented by the simple PCR-ELISA for orienting molecular dermatophyte detection. Fine diagnostics for precise pathogen identification is based on the sequencing of the ITS region of the rDNA. The DermaGenius^®^ Resistance multiplex RT-PCR was also used as part of a method comparison.

*T. mentagrophytes* ITS genotype VIII (*T*. *indotineae*) cannot be clearly sequenced using conventional mycological techniques. ITS sequencing is necessary in all cases. Unfortunately, it must be noted that there is currently no local capacity for this diagnosis in Bangladesh.

### 4.4. Resistance Testing

In vitro susceptibility testing using the breakpoint agar dilution method revealed that 62% of *T. mentagrophytes* ITS genotype VIII (*T*. *indotineae*) strains were terbinafine-resistant. The percentage is also consistent with the terbinafine resistance of isolates of *T. mentagrophytes* ITS genotype VIII isolated in Germany [25,46]. In the large study on terbinafine resistance of *T. mentagrophytes* ITS genotype VIII in India in 2017/19, an even higher percentage of terbinafine-resistant isolates of 66.7% to 76% was found. In addition, 27.3% to 57.1% of Indian *T. rubrum* strains were also terbinafine-resistant [36]. In cases of recurrent dermatophytosis, terbinafine is used repeatedly, which can lead to resistance of the dermatophytes if used uncontrolled over a long period of time [50]. In the Asian region, topical combination preparations are also often used that contain a potent topical glucocorticoid and several antimicrobial agents [46]. These so-called combo creams or cocktail creams predominantly contain clobetasol propionate as a class IV topical glucocorticoid. One reason for the widespread and long-term use of combination preparations is the price, which is significantly lower than that of topical antifungal monopreparations [51]. Additionally, the combo creams are available without a doctor’s prescription and are recommended and sold in over-the-counter (OTC) pharmacies. Terbinafine has no effect on the most strains of *T. mentagrophytes* ITS genotype VIII (*T. indotineae*) in chronic, recurrent forms of tinea, either applied topically or taken orally. Approximately 60%, sometimes up to over 70%, of the isolates show resistance to terbinafine in vitro [36,46,52]. At least 38% of all *T. mentagrophytes* ITS genotype VIII (*T. indotineae*) strains tested were sensitive to terbinafine in vitro. In those patients with in vitro sensitive *T. mentagrophytes* ITS genotype VIII (*T. indotineae*) isolates, therapy with terbinafine may be attempted. However, an alternative treatment, usually itraconazole, should be considered.

In ten terbinafine-sensitive cases (patient nos. 13, 28, 30, 36–38, 73, 77, 78, 93, 97), oral terbinafine was taken in the past without itraconazole or voriconazole and a relapse occurred. It is not essential that patients who have previously received terbinafine/itraconazole therapy also develop terbinafine resistance in vitro. This is especially because the *T. mentagrophytes* ITS genotype VIII (*T*. *indotineae*) strains can also be acquired by transmission from other individuals, with or without terbinafine resistance. Repeated relapses and failure of treatment with oral antifungals are typical of *T. mentagrophytes* ITS genotype VIII (*T*. *indotineae*) infections. These repeated relapses are certainly not only due to antifungal resistance but also to the virulence and altered biological behavior of this new dermatophyte species.

Itraconazole from the azole group was tested as the second antimycotic. About 28% of all *T. mentagrophytes* ITS genotype VIII (*T*. *indotineae*) isolates were resistant to itraconazole in vitro. The ECOFF for itraconazole is 0.25 µg/mL [36,37]. In this practical scenario of clinical non-response to terbinafine, the unrestricted use of voriconazole for dermatophytosis is a new phenomenon in Bangladesh. In the series of chronic recalcitrant dermatophytosis, at least 50% of patients had taken terbinafine orally in the past, 19% had taken voriconazole, and 20% had taken itraconazole with or without prescription. This widespread use of voriconazole for superficial fungal infection may pose a threat of resistance in the future.

There is a good correlation between the result of the breakpoint test, the detection of in vitro terbinafine resistance, and the treatment failure of terbinafine in patients [53,54]. The results of the breakpoint test of *T. mentagrophytes* ITS genotype VIII (*T*. *indotineae*) correlated very well with treatment failure in tinea corporis caused by this pathogen, both with regard to oral therapy and topical application of terbinafine [53,54]. The breakpoint test has also proven to be plausible and clinically relevant in the resistance testing of *T. rubrum*. *T. rubrum* isolates resistant in the breakpoint test do not respond to terbinafine therapy in patients [55,56].

### 4.5. Terbinafine Resistance Due to Mutations in Squalene Epoxidase

According to Yamada et al. (2017) [35], there is a direct connection between mutations in the *SQLE* gene and resistance to terbinafine. A mutation analysis using sequencing demonstrated that 68 of a total of 79 isolates had mutations in this gene. However, only 49 isolates were also resistant to terbinafine. The mutation analysis was also carried out using the DermaGenuis^®^ Resistance Kit. However, this test kit only detects the mutations at positions 393 and 397. These are the amino acid substitutions Leu393Ser, Leu393Phe, Phe397Leu, and Phe397Ile. A total of 46 of the terbinafine-resistant patients were diagnosed, using both multiplex real-time PCR and sequencing to detect point mutations at positions 393 and 397. The Ala448Thr mutation does not affect resistance to terbinafine. In contrast, there appears to be an association with itraconazole resistance [57]. The remaining four terbinafine-resistant isolates showed the mutations Ala448Thr and Ser436Ala. Only a single strain that was resistant to both terbinafine and itraconazole showed the Ala448Thr mutation. The remaining 16 isolates with this mutation showed itraconazole resistance in eight cases and a sensitive reaction to the antifungal in eight cases. These results support the statement that the mutation may cause an increased likelihood of itraconazole resistance, but this does not necessarily lead to resistance. The final mutation Asn429Asp is a mutation that is not responsible for resistance. No resistance was to be expected from the sequencing analysis of the isolates without mutations. This occurred in 91% of these samples. One sample nevertheless showed resistance to itraconazole. Since this is not caused by a mutation in squalene epoxidase, this result is also in line with expectations. For all other samples that could not be sequenced due to missing cultures, a comparison and evaluation of the results of the resistance kit with the specific point mutations was not possible.

### 4.6. Itraconazole Resistance Due to Mutations in the ERG11 Gene

The starting point for this analysis was the publication by Burmester et al. (2022) [41], which showed point mutations in the *Erg11B* gene in *T. mentagrophytes* ITS genotype VIII (*T. indotineae*) isolates obtained from patients at the Jena University Hospital. As in other fungi, the genome of *T. mentagrophytes* ITS genotype VIII (*T. indotineae*) encodes two putative copies of Erg11 that encode sterol 14-α demethylases, named A and B due to their similarity to homologous A and B copies of *Aspergillus fumigatus* [18]. Fungal *Erg11* genes belong to the cytochrome P450 containing protein superfamily 51 and are therefore also synonymously referred to as Cyp51. The two Erg11 proteins of *A. fumigatus* differ in their substrate specificity and *Erg11B* (syn. Cyp51B) only converts eburicol and was unable to use lanosterol as substrate [58]. Eburicol is formed from lanosterol by the action of the C-24 methyltransferase encoded by Erg6 [59] and accumulation of eburicol has toxic effects in *A. fumigatus* [60]. In *A. fumigatus*, deletion mutants of one *Erg11* gene copy have a viable phenotype, showing that each gene can replace the function of the second copy [61]. Erg11-related azole resistance depends on overexpression of *Erg11* genes or point mutations leading to amino acid exchanges of Erg11 protein sequences [62]. Another Erg11-independent resistance mechanism in fungi is the efflux of azoles due to increased expression of multiple drug resistance (MDR) transporters or major facilitator superfamily (MFS) transporters [63]. The influence of the MDR3 transporter on the transport of voriconazole and itraconazole has been shown for *T. rubrum* [64]. Nevertheless, *T. mentagrophytes* ITS genotype VIII (*T. indotineae*) disrupting MDR3 and its parental strains overexpressing MDR3 show little difference in azole susceptibility [65]. Genome analysis of *T. mentagrophytes* ITS genotype VIII (*T. indotineae*) shows a correlation of multiple *Erg11B* copies as tandem repeats with overexpression of *Erg11B* [65]. Interestingly, two *Erg11B*-overexpressing mutant strains showing itraconazole resistance also contain the Ala448Thr mutation in the squalene epoxidase protein sequence. One of the itraconazole-resistant Ala448Thr mutant strains contains only one *Erg11B* copy in combination with *Erg11B* overexpression [65]. Therefore, the high number of Ala448Thr mutant strains showing itraconazole resistance in this study may also be the result of *Erg11B* overexpression due to several mechanisms. Interestingly, in a previous study, an Ala448Thr mutant strain showed multiple azole resistance in combination with *Erg11B* wild-type sequences [18], indicating a resistance mechanism independent of *Erg11B* point mutations.

Point mutations in Erg11 genes that result in amino acid exchanges mediate resistance to specific azoles due to their chemical nature [66]. For example, the Erg11A mutant Tyr121Phe from *A. fumigatus* shows resistance to voriconazole, which belongs to the short-chain azoles [66]. *Erg11B* point mutations in this study affected similar amino acid positions as in a previous study [18]. *Erg11B* mutations Asp441Gly, Gly443Glu, Tyr444Cys, and Tyr444His were found in both studies. However, several of these mutations do not show a clear phenotype against azoles used in medical therapy [18]. Only *Erg11B* Ala230Thr shows an association with resistance against sertaconazole nitrate [18]. Interestingly, plant pathogenic fungi show comparable mutations in Erg11 as found in *Erg11B* of *T. mentagrophytes* ITS genotype VIII (*T. indotineae*). The wheat pathogen *Zymoseptoria* (*Z.*) *tritici* (tel. *Mycosphaerella graminicola*) shows small deletions of delta Tyr459/Gly460 [67], which corresponds to Tyr442/Gly443 in the wild-type *Erg11B* sequences of *T. mentagrophytes* ITS genotype VIII (*T. indotineae*) presented here. In addition, the *Z. tritici* mutations Tyr461His and Tyr461Ser [67] correspond to the mutations Tyr444His and Tyr444Ser in *T. mentagrophytes ITS genotype* VIII (*T. indotineae*). Erg11 mutants in *Z. tritici* show increased resistance to azole used in agriculture as cyproconazole [67]. Other phytopathogenic fungi show similar mutations, for example, the banana pathogen *Mycosphaerella fijiensis* carries Erg11 mutations Tyr463His [68] that correspond to *T. mentagrophytes* ITS genotype VIII (*T. indotineae) Erg11B* Tyr444His. Other *Mycosphaerella fijiensis* mutations show several other tyrosine substitutes in Erg11 protein positions 461 and 463 [68]. The mutations correlate with loss of sensitivity to propiconazole [68].

The high frequency of *Erg11B* mutations in *T. mentagrophytes* ITS genotype VIII (*T. indotineae*) leading to amino acid exchanges shows that there is a high selection pressure for the evolution of *Erg11B* due to environmental changes. This also explains the increase in other resistance mechanisms such as overexpression of *Erg11B* [47]. The fact that no direct relation with medically used azoles was observed for several *Erg11B* mutants suggests that azoles widely used in agriculture might have an influence on the development of *Erg11B* in *T. mentagrophytes* ITS genotype VIII (*T. indotineae*). It is important to elucidate the role of these evolutionary tendencies to understand the fungal repertoire of resistance mechanisms in the future. The unique genotypes of each isolate suggest that the resistant strains evolved independently at different times. The challenge for future diagnostics is therefore not only to determine the species subtypes but also to analyze genes involved in the resistance mechanisms [18].

Resistance to itraconazole could not be explained by mutations found in the *ERG11B* gene.

## 5. Conclusions

In Bangladesh, infection with *T. mentagrophytes* ITS genotype VIII (*T. indotineae*) has become a public health issue with potentially global ramifications. Here, 62% of samples showed resistance to terbinafine, making oral itraconazole the most effective drug currently available. However, itraconazole resistance is also increasing. The scenario is identical in India and Bangladesh.

## Figures and Tables

**Table 1:** List of strains and genotypes used to construct the dendrogram, including the accession number of the National Center for Biotechnology Information (NCBI) in Bethesda, MD, USA. The sequences obtained in the present study are marked in bold. II* is a subgroup of genotype II. III* is a subgroup of genotype III.

Strain Number, Moelbis Lab	Collection	ITSrDNA–Genebank NCBI	tef1-α–Genebank NCBI	Species
600270 19	-	OM951137	Moelbis lab	*T. interdigitale* ITS genotype I
208223 17	DSM 108620	MK447595	MK460538	*T. interdigitale* ITS genotype I
600086 21	-	OM951149	Moelbis lab	*T. interdigitale* ITS genotype I
200070 17	DSM 108,621	MK447596	MK460539	*T. interdigitale* ITS genotype II
600283 19	-	OM951146	600283 19	*T. interdigitale* ITS genotype II
212583 21	-	OM951143	Moelbis lab	*T. interdigitale* ITS genotype II
250016 18	-	MN886818	MN886231	*T. mentagrophytes* ITS genotype II*
212063 17	DSM 108905	MK630684	MK751367	*T. mentagrophytes* ITS genotype II*
208787 21	-	OM951152	Moelbis lab	*T. mentagrophytes* ITS genotype III
200002 16	DSM 103451	KX866689	MK460540	*T. mentagrophytes* ITS genotype III
217704 15	DSM 108630	MK450325	MK460541	*T. mentagrophytes* ITS genotype III
217907 15	DSM 108628	MK447605	MK460542	*T. mentagrophytes* ITS genotype III*
218893 16	DSM 108629	MK447604	MK460543	*T. mentagrophytes* ITS genotype III*
900120 17	DSM 108632	MK447606	MK460544	*T. mentagrophytes* ITS genotype III*
-	UKJ 594/19	MN064822.1	-	*T. mentagrophytes* ITS genotype VIII *T. indotineae*
204532 20	-	MT333242	MT340525	*T. mentagrophytes* ITS genotype VIII *T. indotineae*
220575 19	-	MT330287	MT340521	*T. mentagrophytes* ITS genotype VIII *T. indotineae*
214174 19	DSM 110675	MT330289	MT340511	*T. mentagrophytes* ITS genotype VIII *T. indotineae*
218160 18	DSM 108899	MT330253	MT340503	*T. mentagrophytes* ITS genotype VIII *T. indotineae*
216377 17	DSM 108902	MT330249	MT340500	*T. mentagrophytes* ITS genotype VIII *T. indotineae*
214677 16	DSM 108903	MT330252	MT340499	*T. mentagrophytes* ITS genotype VIII *T. indotineae*
-	CBS 130940	-	KM678173.1	*T. mentagrophytes* ITS genotype VIII *T. indotineae*
**600158 22**	**-**	**PQ216375**	**PQ232479**	***T. mentagrophytes*** **ITS genotype VIII *T. indotineae***
**600145 22**	**-**	**PQ216374**	**PQ232478**	***T. mentagrophytes*** **ITS genotype VIII *T. indotineae***
**600121 22**	**-**	**PQ216373**	**PQ232477**	***T. mentagrophytes*** **ITS genotype VIII *T. indotineae***
**600115 22**	**-**	**PQ216372**	**PQ232476**	***T. mentagrophytes*** **ITS genotype VIII *T. indotineae***
**600103 22**	**-**	**PQ216371**	**PQ232475**	***T. mentagrophytes*** **ITS genotype VIII *T. indotineae***
215003 16	DSM 108624	MK450324	MK467449	*T. mentagrophytes* ITS genotype VII
210363 16	DSM 108625	MK450323	MK467450	*T. mentagrophytes* ITS genotype VII
218904 16	DSM 108622	MK450322	MK467448	*T. mentagrophytes* ITS genotype VII
204543 17	DSM 108626	MK447609	MK467445	*T. mentagrophytes* ITS genotype IV
200602 17	DSM 108631	MK447607	MK467447	*T. mentagrophytes* ITS genotype IV
200617 17	DSM 108627	MK447608	MK467446	*T. mentagrophytes* ITS genotype IV
112636 16	-	PQ248103	Moelbis lab	*T. quinckeanum*

**Table 2:** List of the strains from Bangladesh deposited at the database of the National Center for Biotechnology Information (NCBI) in Bethesda, MD, USA.

Species	Country	Strain Number, Moelbis Lab	Year	Genebank NCBI
*T. mentagrophytes* ITS genotype VIII *T. indotineae*	Bangladesh	600103/22	2022	PQ216371 (ITS) PQ232475 (*tef1-α*)
*T. mentagrophytes* ITS genotype VIII *T. indotineae*	Bangladesh	600115/22	2022	PQ216372 (ITS) PQ232476 (*tef1-α*)
*T. mentagrophytes* ITS genotype VIII *T. indotineae*	Bangladesh	600121/22	2022	PQ216373 (ITS) PQ232477 (*tef1-α*)
*T. mentagrophytes* ITS genotype VIII *T. indotineae*	Bangladesh	600145/22	2022	PQ216374 (ITS) PQ232478 (*tef1-α*)
*T. mentagrophytes* ITS genotype VIII *T. indotineae*	Bangladesh	600158/22	2022	PQ216375 (ITS) PQ232479 (*tef1-α*)
*Trichophyton rubrum*	Bangladesh	600173/22	2022	PQ216376 (ITS) PQ232480 (*tef1-α*)

**Table 3:** List of occupation (n = 95).

Occupation	Number
Housewife	33
Student	25
Service	19
Businessman	5
Peasant	3
Industry worker	2
Teacher	2
Laborer	1
Govt. service	1
Shopkeeper	1
Tailor	1
Retired	1
No job	1

**Table 4:** History of previous FDCs or intramuscular corticosteroid injection. Specifying multiple preparations was possible.

History of Previous FDCs	Number
Clobetasol + ofloxacin + ornidazole + terbinafine	33
Econazole + triamcinolone	20
Miconazole + hydrocortisone	14
Triamcinolone injection	6
Clobetasol	6
Betamethasone	2
Mometasone	1
Triamcinolone	1
Not sure	9
No	6
Yes	1

**Table 5:** Number of dermatophytes found as well as terbinafine- and itraconazole-resistant and -sensitive strains (n = 76).

	Cultures Grown	Terbinafine (%)		Itraconazole (%)	
		-Resistant	-Sensitive	-Resistant	-Sensitive
*T. mentagrophytes*/*T. interdigitale*	76	49 (64%)	27 (36%)	21 (28%)	55 (72%)
Total	76	76 (100%)		76 (100%)	

**Table 6:** Association between terbinafine and itraconazole resistance and point mutations.

	Terbinafine-Resistant + Itraconazole-Resistant	Terbinafine-Resistant + Itraconazole-Sensitive	Terbinafine-Sensitive + Itraconazole-Resistant	Terbinafine-Sensitive + Itraconazole-Sensitive	Total
F397L	3	17	0	0	20
A448T	1	0	10	6	17
L393S	2	17	0	1	20
S436A	2	1	0	2	5
F397I	0	1	0	0	1
L393F	0	1	0	0	1
N429D	0	0	0	1	1
Double mutation F397L and A448T	3	1	0	0	4
Total mutations	11	38	10	10	69
No mutation (wild type)	0	0	0	7	7
Total	11	38	10	17	76

**Table 7:** Results of resistance testing using the DermaGenius^®^ Resistance Kit.

	Mutation Identification	Pathogen Identification (%)	
		*T. interdigitale/T. mentagrophytes*	*T. rubrum*
Wild type	38 (38.4)	34 (34.3)	4 (4.1)
Mutant	61 (61.6)	61 (61.6)	0 (0.0)
Total	99 (100)	95 (95.9)	4 (4.1)

**Table 8:** Association between point mutations in the *Erg11B* gene and resistance to itraconazole based on all samples examined.

Mutation	Base Exchange in the *Erg11* Gene	Itraconazole Testing	Number	Percent (%)
D441Y	GAT → TAT	Sensitive	4	5.0
D441G	GAT → GGT	Sensitive	1	1.3
G443E	GGA → GAA	Resistant	1	1.3
		Sensitive	1	1.3
G443R	GGA → AGA	Sensitive	1	1.3
Y444H	TAC → CAC	Resistant	2	2.5
		Sensitive	28	35.4
Y444C	TAC → TGC	Resistant	2	2.5
		Sensitive	3	3.8
Y444S	TAC → TCC	Sensitive	4	5.1
G445S	GGT → AGT	Resistant	2	2.5
		Sensitive	1	1.3
G445D	GGT → GAT		1	1.3
Total mutations			51	64.6
No mutations		Resistant	15	19.0
		Sensitive	13	16.4
Total			79	100

**Table 9:** Association between itraconazole resistance in vitro and mutations in the *Erg11B* gene in *T*. *mentagrophytes ITS genotype* VIII (*T. indotineae*) strains (n = 79).

Mutation	Mutation Present (%)	Mutation Absent (%)	Total (%)
Itraconazole-resistant	7 (8.9)	15 (19.0)	22 (27.9)
Itraconazole-sensitive	44 (55.7)	13 (16.4)	57 (72.1)
Total	51 (64.6)	28 (35.4)	79 (100)

**Table 10:** Comparison of mutations in the *Erg11B* gene with the Ala448Thr mutation in the *SQLE* gene (n = 79).

Mutation (*Erg11B*)	Ala448Thr (*SQLE*)	Number	Percent (%)
D441Y	Absent	4	5.1
D441G	Absent	1	1.3
G443E	Absent	2	2.5
G443R	Absent	1	1.3
Y444H	Absent	30	37.8
Y444C	Absent	5	6.3
Y444S	Absent	4	5.1
G445S	Absent	3	3.8
G445D	Present	1	1.3
None	Present	18	22.8
	Absent	10	12.7
Total		79	100

## Data Availability

The raw data supporting the conclusions of this article will be made available by the authors on request.

## Supplementary Materials

The following supporting information can be downloaded at: https://www.mdpi.com/article/10.3390/jof10110768/s1, Table S1: Clinical data and resistances of patients from Bangladesh.
